# Bioinformatics analysis and genetic polymorphisms in genomic region of the bovine *SH2B2* gene and their associations with molecular breeding for body size traits in qinchuan beef cattle

**DOI:** 10.1042/BSR20192113

**Published:** 2020-03-12

**Authors:** Sayed Haidar Abbas Raza, Rajwali Khan, Linsheng Gui, Nicola M. Schreurs, Xiaoyu Wang, Chugang Mei, Xinran Yang, Cheng Gong, Linsen Zan

**Affiliations:** 1 College of Animal Science and Technology, Northwest A&F University, Yangling, Shaanxi, 712100, P.R. China; 2 State Key Laboratory of Plateau Ecology and Agriculture, Qinghai University, Xining, Qinghai Province 810016, People’s Republic of China; 3 Animal Science, School of Agriculture and Environment, Massey University, 4442 Palmerston North, New Zealand; 4 National Beef Cattle Improvement Center, Northwest A&F University, Yangling, 712100 Shaanxi, P.R. China

**Keywords:** body measurement, polymorphism, Qinchuan cattle, SH2B2 Gene

## Abstract

The Src homology 2 B 2 (*SH2B2*) gene regulate energy balance and body weight at least partially by enhancing Janus kinase-2 (JAK2)-mediated cytokine signaling, including leptin and/or GH signaling. Leptin is an adipose hormone that controls body weight. The objective of the present study is to evaluate the association between body measurement traits and *SH2B2* gene polymorphisms as responsible mutations. For this purpose, we selected four single-nucleotide polymorphisms (SNPs) in *SH2B2* gene, including two in intron 5 (g.20545A>G, and g.20570G>A, one synonymous SNP g.20693T>C, in exon 6 and one in intron 8 (g.24070C>A, and genotyped them in Qinchuan cattle. SNPs in sample populations were in medium polymorphism level (0.250<PIC<0.500). Association study indicated that the g.20570G>A, g.20693T>C, and g.24070C>A, significantly (*P* < 0.05) associated with body length (BL) and chest circumference (CC) in Qinchuan cattle. In addition, H4H3 and H5H5 diplotype had highly significantly (*P* < 0.01) greater body length (BL), rump length (RL), and chest circumference (CC) than H4H2. Our investigation will not only extend the spectrum of genetic variation of bovine *SH2B2* gene, but also provide useful information for the marker assisted selection in beef cattle breeding program.

## Introduction

To get long-term improvement in growth and key carcass characteristics that have economic importance, selective breeding is used but, it can be difficult to get efficient genetic gain using traditional breeding methods due to long periods required to finish progeny in order to get information on performance [1,2]. Marker-assisted selection (MAS) for improving desirable traits is powerful and efficient [3,4]. Based on the biological function, the genes that are involved in meat quality traits or body measurements of production animals can be identified [5,6]. Qinchuan cattle used in this research are an indigenous breed in China, and are known to have good meat quality, adaptability in farming systems, and desirable physical features [7–9]. So, it would be valuable to understand the biological function of genes that are associated with carcass characteristics and body or growth traits [10]. In the process of livestock breeding, body measurement and meat quality traits are used as a tool to assess the economic value of animals. It has been demonstrated that many genes are related to, meat production [11], growth [12], and meat quality traits [13]**.**

The SH2B family has three members (*SH2B1, SH2B2*, and *SH2B3*) that contain conserved dimerization (DD), pleckstrin homology, and SH2 domains. Previously, SH2B2 that categorized as an adapter protein with a PH and SH2 domain (APS) is a member of the Src homology 2 B (SH2B) and has a conserved structure of a N-terminal dimerization domain (DD), a central pleckstrin homology (PH) domain, and a C-terminal Src homology 2 (SH2) domain [14]. *SH2B2* may regulate energy balance and body weight partially by enhancing Janus kinase-2 (JAK2)-mediated cytokine signaling, including leptin and growth hormone signaling. In cultured cells, *SH2B2* binds via its SH2 domain to JAK2, potentiating JAK2 activation [15,16] and also binds to the insulin receptor, promoting the insulin signaling pathway [17,18]**.** Moreover, *SH2B2* didn’t affect insulin receptor numbers or insulin receptor turnover both *in vivo* and *in vitro*; however, *SH2B2* increased insulin sensitivity in mice [19]. Therefore, *SH2B2* has activity in mediating the insulin-stimulated activation of the c-Cb1/CAP/TC10 pathway that appears to play an important role in regulating glucose uptake in cultured adipocytes [20]. *SH2B2* is expressed in multiple tissues, including targets of insulin, GH, and leptin (e.g. the brain, adipose tissue, and skeletal muscle) [14,21,22]. *SH2B2*, on the other hand, are known as negative regulators of B-cell proliferation [23,24] and the mRNA expression in Qinchuan beef cattle that we have detected, we found that there is a high expression of *SH2B2* not only in fat but also in kidney and other splanchnic tissues, which might bring some change about animal traits. Thus, we hypothesized that *SH2B2* might be associated with conformation and carcass traits on beef cattle.

There has been a lack of information about the association of bovine *SH2B2* genotypes with body measurement traits in Qinchuan cattle**.** Therefore, the present study was designed to identify the effects of polymorphisms on *SH2B2* in 468 individual Chinese Qinchuan cattle by using Real-time PCR to analyze tissue expression patterns and establishing a correlation between the bovine *SH2B2* gene mutations and body measurements to identify associated quantitative traits for the benefit of cattle breeding and genetics.

## Materials and methods

### Bioinformatics analyses

The bioinformatics techniques were used for the measurement of degree of conservation and biological evolution of *SH2B2* protein in different species. The amino acid sequences of SH2B2 gene were acquired from NCBI (www.ncbi.nlm.nih.gov/protein) for *Bos taurus* (XP_024841048.1), *Homo sapiens* (NP_066189.3), *Ovis aries* (XP_027817506.1), *Mus musculus* (NP_061295.2), *Ovis aries* (XP_023511156.1), *Bubalus bubalis* (NP_001277771.1), *Equus caballus* (NP_001265704.1), *Gallus gallus* (XP_015151487.1), *Felis catus* (XP_023102534.1), *Cavia porcellus* (XP_013008193.1) *Oryctolagus cuniculus* (XP_008251155.1) *Macaca mulatta* (XP_014990031.1) *Canis lupus familiaris* (XP_005621054.1). Sequence similarity between bovine *SH2B2* protein and its homologue was performed. The Jalview Jalview software’s were used for multiple sequence alignment (http://www.jalview.org/). The analysis of protein structure and function, the motifs were searched, and conserved domains were identified through the online MEME suite website [25].

### Feeding and management of Qinchuan cattle and phenotypic data collection

Total 468 female cows (non-pregnant) of Qinchuan breed cattle maintained at the experimental farm of National Beef Cattle Improvement Research Centre, Yangling, China were selected for conducting this research study. All the experimental animals were aged between 18 and 24 months of age and were randomly selected from *Qinchuan* cattle breeding populations, the subject animals were fed a total mixed ration (TMR), containing 25% concentrate and 75% roughages of dry straw and corn silage, and water was offered ad libitum. The feeding was offered based on NRC standards (Nutrient Requirement of Beef Cattle) [26]. Moreover, all animals were kept under uniform management system with same environment (i.e. temperature and humidity) in the shed. Animals were stunned with a captive bolt and slaughtered through exsanguination, then the collected samples were snap-frozen in liquid nitrogen for tissue RNA isolation. All samples were stored at −80°C until subsequent analyses.

Genomic DNA were extracted from blood samples (collected from the jugular vein) using a blood DNA Kit (OMGAM Bio-Tek, Doraville, U.S.A.). The DNA content was estimated spectrophotometrically, and diluted to 50 ng/μl. Meanwhile, body measurement traits (BMTs), including body length (BL), withers height (WH), hip height (HH), rump length (RL), hip width (HW), chest depth (CD), and chest circumference (CC), were measured as described previously [10] for association analyses.

### Primer design and PCR conditions

There primers to amplify of the bovine *SH2B2* gene were designed based on NCBI database (GenBank accession number NC_037352.1) CDS region of ∼1949 kb. First, we mixed 468 DNA samples with equal molar ratio to constitute a DNA pool [27]. Then, DNA from 468 Qinchuan cattle were performed for PCR using Primer v5.0 software (PREMIER Biosoft International, California, U.S.A.). Primers, annealing temperature, region, and fragment sizes are shown in Table 1. The PCR was carried out in a total volume of 20 μl containing 50 ng DNA, 10 pM of each primer, 0.20 mM dNTP, 2.5 mM MgCl_2_ and 0.5 U Taq DNA polymerase (TaKaRa, Dalian, China). The PCR protocol was 5 min at 95°C; 35 cycles of 30 s at 94°C, 35 s at corresponding temperature, 40 s at 72°C, and a final extension step at 72°C for 10 min. The digested products were detected by electrophoresis technique in a 0.8% agarose gels containing 0.5 μg of ethidium bromide/ml. The PCR products were sequenced through Sangon (Shanghai, China) to screen for polymorphisms. All sequences were checked using Seq Man (DNASTAR, Inc., U.S.A.) software, and the SNPs were identified.

**Table 1:** Primers used in the present study

Name	Function	Primer Sequence (5′ to 3′)	*T*_m_ (°C)	Product length	Amplified region
SH2B2	qPCR	TTTCTCACGGTCTCGGTTCC	58	271 bp	99–369
		CCGGAGGCTCTCCCC			
GAPDH	Reference	CCAACGTGTCTGTTGTGGAT	61	80 bp	778–857
		CTGCTTCACCACCTTCTTGA			
Primer A	SNP detection	GTTGGGCTTTCTTGCCTTCG	60	594 bp	Intron 5
		CCCTTTCCGTGAGTATTTTCTACC			
Primer B	SNP detection	CCTGTTCCCTCATTTGATACATTCTC	58	496 bp	Exon 6
		TGTTTCCCTTTGTGCCTTAGGTATT			
Primer C	SNP detection	CCTTCAAACGCACTTGCCAATC	60	574 bp	Intron 8
		GCACTTTCACTCACCGCTCCC			

### Analysis of mRNA relative expression and real-time PCR

The eight tissue specimens, including muscle, rumen, fat, abomasum, heart, spleen, kidney, and small intestine were collected from three female Qinchuan cattle aged 18 months old (*n* = 3). The RNA was extracted from each tissue sample using the Trizol reagent kit (TIANGEN, China), and was subjected to reverse transcription (RT) to obtain the corresponding cDNA (TaKaRa, Dalian, China). After collection from the tissue, samples were preserved in liquid nitrogen and were transferred immediately in frozen form to the molecular laboratory for the extraction of total RNA. The total RNA was extracted from the tissue using TRIzol™ Reagent (Invitrogen, ThermoFisher Scientific, Inc. U.S.A.). Data were normalized to the geometric mean of GAPDH (GenBank Accession no. NM_001034034) used as endogenous control genes. The primers used are given in Table 1. Real-time quantitative PCR was performed using the ABI 7500 RT-PCR system (Applied Biosystems, NY, U.S.A.) with the reagent TB Green Premix Ex Taq II (Takara, Kusatsu, Japan), calculated using the 2-ΔΔCt method [28].

### Statistical analysis

Gene and allelic frequency of four SNPs were determined and Hardy–Weinberg equilibrium (HWE) were calculated through χ2 test via the PopGene software [29]. Linkage disequalibrium (LD) tests containing value of D’ and γ2 were evaluated through HAPLOVIEW (Version 3.32) (Barrett 2005). Other population genetic data, like gene heterozygosity (He) or polymorphism information content (PIC), was statistically analyzed according to established methods [30]. The haplotype data were analyzed by the website tool: SHEsis software [31,32].

Analysis of associations between the genotypes of SNPs and body measurement traits was carried out with the GLM procedure, using SPSS software (version 13.0) by the following formula: Yij = u+Gi+Ai+Eijk

Where Yij was the traits measured on each of the individual cattle, μ was the overall population mean for the traits, Gi was the fixed effect associated with the genotype, Ai was the fixed effect due to the age and Eijk was the standard error.

The mean relative mRNA expression level of SH2B2 gene in different tissues and at different age groups was analyzed by ANOVA using computer software SAS (version 8.1).

## Results

### Polymorphisms and genetic diversity

Four polymorphism sites in SH2B2 gene, including (snp1 g.20545A>G, snp2 g.20570G>A, snp3 g.20693T>C, and snp4 g.24070C>A, were identified by sequencing. Genotype and allele frequency for the 4 loci are shown in (Table 2). An allele of g.20545A>G, g.20570G>A and g.24070C>A, and T allele of g.20693T>C was predominant at the four SNPs. The PIC value is an effective variability to assess the genetic diversity from different loci of candidate gene. Our results showed that those SNPs were in medium polymorphism level (0.250<PIC<0.500). By χ^2^ test, the genotypic distributions of g.20570G>A, and g.20693T>C, differed significantly from Hardy–Weinberg equilibrium (*P* < 0.05) (see in Table 3). Genetic parameters including genotype and allele frequencies were calculated from total 468 cattle heads of Qinchuan breed.

**Table 2:** Genotype frequencies (%) of the SH2B2 gene for the SNPs

Site	Sample	Genotypic frequency			Allele frequency		χ2 (HW*)	PIC	He	Ne
g.20545A>G	468	AA	AG	GG	A	G				
		0.5427	0.3697	0.0876	0.7276	0.2724	2.1342	0.3179	0.3964	1.6568
g.20570G>A	468	GG	AG	AA	G	A				
		0.2137	0.2885	0.4979	0.3579	0.6421	64.8993	0.3540	0.4596	1.8505
g.20693T>C	468	TT	TC	CC	T	C				
		0.5363	0.2821	0.1816	0.6774	0.3226	58.8840	0.3416	0.4371	1.7765
g.24070C>A	468	CC	CA	AA	C	A				
		0.6432	0.3056	0.0513	0.7959	0.2041	1.6492	0.2721	0.3248	1.4811

Note: HW, Hardy–Weinberg equilibrium; χ0.05^2^ = 5.991, χ0.01^2^ = 9.21

**Table 3:** Estimated values of linkage disequilibrium for SNPs bovine SH2B2

SNP	A20545G	G20570A	T20693C	C24070A
g.20545A>G	–	*D* = 0.042	*D* = 0.052	*D* = 0.012
g.20570G>A	*r*^2^ = 0.000	–	*D* = 0.538	*D* = 0.616
g.20693T>C	*r*^2^ = 0.000	*r*^2^ = 0.247	–	*D* = 0.794
g.24070C>A	*r*^2^ = 0.000	*r*^2^ = 0.157	*r*^2^ = 0.343	–

### LD and haplotype analysis

There are two most commonly used indicators for the prediction of linkage disequilibrium (LD). One is *D* and other is *r*^2^. There is a consensus of the researchers that the latter indicator is most commonly used for pair wise measurement of the LD and hence consider less sensitive for the measurement of allelic frequencies than *D*’ [33,34].

**Figure 1:**
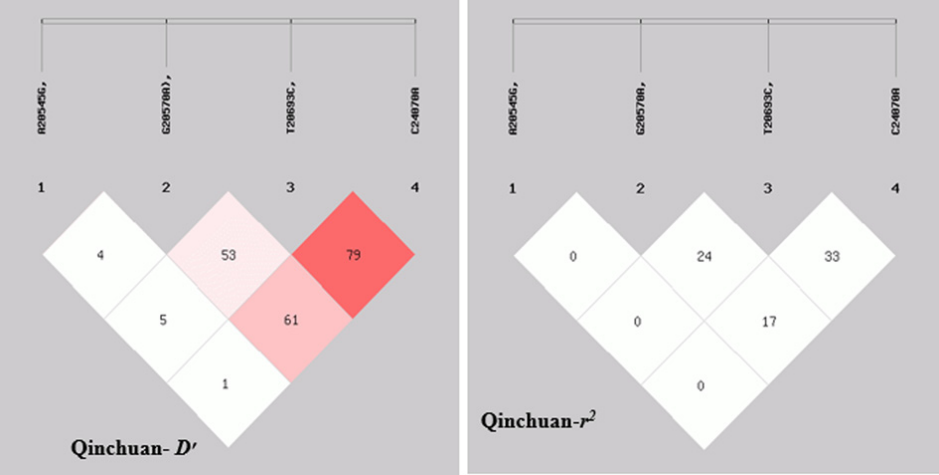
Linkage disequilibrium (LD) plot (*D* and *r*^2^) of four novel SNP loci within the SH2B2 gene in Qinchuan cattle

In the present study, the LD was highest between g.20693T>C, and g.24070C>A, (Table 4). In addition, Hap1 (–AATC–) had the highest haplotype frequencies (33.70%), followed by Hap5 (–GATC–), and Hap2 (–AGCA–) (Figure 1).

**Table 4:** Haplotypes of SH2B2 gene and their frequencies

Haplotype	A20545G	G20570A	T20693C	C24070A	Frequency
Hap1	A	A	T	C	0.337
Hap2	A	G	C	A	0.102
Hap3	A	G	C	C	0.081
Hap4	A	G	T	C	0.084
Hap5	G	A	T	C	0.146

### Effects of single markers/ haplotype combinations on growth traits in Qinchuan cattle

In this paper, four polymorphisms seem to mainly affect bovine body measurement traits (Table 5). At g.20570G>A locus, individuals with genotype GG had higher values than those with GA on BL and CC (*P* < 0.05). At g.20693T>C locus, genotype CC had higher mean values for BL and CC than these with the genotype TT (*P* < 0.05). At g.24070C>A, locus, significant differences of BL, RL and CC were observed between CC and AA genotypes (*P* < 0.05). No significant correlations were observed in the rest of the index for the four SNPs. In Table 6, multiple effects of the four SNPs were evaluated. H_4_H_3_ and H_5_H_5_ diplotype had highly significantly greater BL, RL and CC than H_4_H_2_ (*P* < 0.05), similarly results were found between H_4_H_3_ and H_1_H_1_ (*P* < 0.05).

**Table 5:** Association of different genotypes of SNPs in SH2B2 with body measurement traits

		BL (cm)	WH (cm)	HH (cm)	RL (cm)	HW (cm)	CD (cm)	CC (cm)
g.20545A>G	AA	133.52 ± 0.45	119.93 ± 0.36	123.20 ± 0.41	41.81 ± 0.22	38.79 ± 0.27	58.97 ± 0.34	163.37 ± 0.77
	AG	134.43 ± 0.54	120.87 ± 0.43	123.62 ± 0.41	42.45 ± 0.27	39.69 ± 0.33	59.65 ± 0.41	166.15 ± 0.83
	GG	136.74 ± 1.11	12.55 ± 0.89	124.27 ± 0.85	43.61 ± 0.55	38.83 ± 0.66	59.50 ± 0.85	164.81 ± 1.33
	**P**	**0.064**	**0.091**	**0.245**	**0.060**	**0.250**	**0.208**	**0.490**
g.20570G>A	GG	139.01 ± 0.64^a^	122.62 ± 0.54	125.42 ± 0.53	41.16 ± 0.34	41.16 ± 0.41	61.33 ± 0.52	169.12 ± 1.18^a^
	GA	129.88 ± 0.55^b^	117.86 ± 0.46	121.65 ± 0.52	40.77 ± 0.29	43.35 ± 0.35	57.19 ± 0.45	159.06 ± 1.01^b^
	AA	134.52 ± 0.42^a,b^	120.89 ± 0.35	123.64 ± 0.34	42.36 ± 0.22	39.28 ±0.28	59.58 ± 0.34	165.72 ± 0.77^a^
	**P**	**0.000**	**0.143**	**0.066**	**0.199**	**0.331**	**0.083**	**0.037**
g.20693T>C	TT	134.77 ± 0.43^a^	120.91 ± 0.35	123.73 ± 0.34	42.36 ± 0.22	39.34 ± 0.27	59.62 ± 0.34	165.85 ± 0.76^a^
	TC	135.67 ± 0.44^a^	120.82 ± 0.49	124.00 ± 0.47	42.69 ± 0.31	39.81 ± 0.37	59.79 ± 0.47	165.19 ± 1.06^a^
	CC	129.91 ± 0.74^b^	118.35 ± 0.61	121.77 ± 0.58	40.95 ± 0.38	37.46 ± 0.47	57.40 ± 0.58	159.56 ±1.32^b^
	**P**	**0.030**	**0.372**	**0.297**	**0.114**	**0.532**	**0.164**	**0.006**
g.24070C>A	CC	135.77 ± 0.39^a^	121.37 ± 0.32	124.08 ± 0.31	42.67 ± 0.20^a^	39.81 ± 0.25	60.09 ± 0.31	166.66 ± 0.69^a^
	CA	131.41 ± 0.57^a,b^	118.94 ± 0.46	122.39 ± 0.45	41.62 ± 0.29^a,b^	38.09 ± 0.35	57.99 ± 0.44	160.96 ± 1.00^b^
	AA	129.98 ± 1.40^b^	117.18 ± 1.13	121.85 ± 1.09	39.66 ± 0.71^b^	36.71 ± 0.87	56.58 ± 1.08	158.83 ± 1.77^b^
	**P**	**0.042**	**0.369**	**0.244**	**0.015**	**0.078**	**0.285**	**0.051**

a,b Means with different superscripts are significantly different (*P* < 0.05).

**Table 6:** Associations of haplotypes with growth traits in *Qinchuan* cattle

Hap	BL (cm)	WH (cm)	HH (cm)	RL (cm)	HW (cm)	CD (cm)	CC (cm)
Hap1/1(120)	134.14 ± 0.64^b^	120.56 ± 0.53	123.60 ± 0.48	41.81 ± 0.31^b^	38.88 ± 0.4	59.31 ± 0.5	164.55 ± 1.08^b^
Hap1/5(79)	134.48 ± 0.79^b^	121.25 ± 0.65	123.58 ± 0.59	42.80 ± 0.39^a,b^	40.01 ± 0.49	60.12 ± 0.62	167.43 ± 1.33^a,b^
Hap4/2(16)	132.94 ± 1.76^b^	119.06 ± 1.44	122.97 ± 1.3	41.75 ± 0.86^b^	38.69 ± 1.08	58.94 ± 1.37	164.13 ± 2.96^b^
Hap4/3(28)	140.36 ± 1.33a	122.57 ± 1.09	124.5 ± 0.98	44.29 ± 0.65^a^	41.5 ± 0.82	61.68 ± 1.04	168.11 ± 2.24^a^
Hap4/4(8)	138.38 ± 2.49^a^	121.25 ± 2.04	124.56 ± 1.84	42.88 ± 1.22^a,b^	41 ± 1.53	60.75 ± 1.94	169.5 ± 4.18^a^
Hap5/5(21)	139.22 ± 1.54^a^	122.26 ± 1.26	125.02 ± 1.14	44.29 ± 0.75^a^	39.57 ± 0.95	60.57 ± 1.2	167.9 ± 2.58^a,b^
P	0.032	0.353	0.270	0.029	0.078	0.223	0.013

a,b Means with different superscripts are significantly different (*P* < 0.05).

### SH2B2 gene expression profile

**Figure 2:**
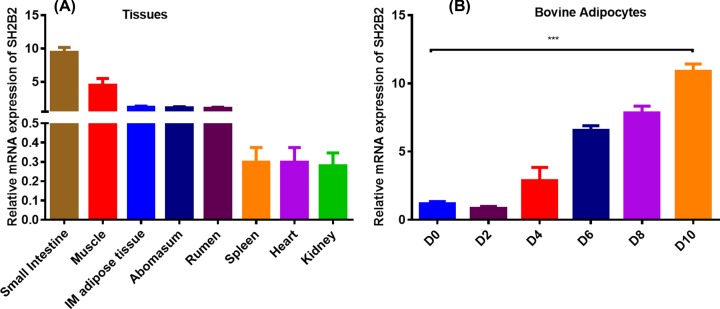
Expression level of *SH2B2* gene (**A**) The mRNA expression level of the SH2B2 gene in different tissues. (**B**) The mRNA expression level of the *SH2B2* gene in different time points of adipocytes differentiation. Glyceraldehyde 3-phosphate dehydrogenase (GAPDH) was used as the house keeping gene One-way ANOVA was used for statistical analysis. Asterisks indicate significant variations. ***P < 0.01.

Results for *SH2B2* relative expression levels in each tissue were shown in Figure 2A,B. The SH2B2 has a wide tissue distribution in the bovine tissues examined, with expression in small intestine, muscle and fat being the highest. The mRNA expression level in abomasum, rumen, and spleen tissues were second highest. The *SH2B2* was expressed only slightly in the heart and kidney tissue. There could be both direct and indirect relationships between body size and metabolism due physiological modulation from *SH2B2*. We also analyzed expression level of *SH2B2* in bovine preadipocytes and adipocytes at different time points (Figure 6B). Expression level of *SH2B2* in differentiated adipocytes was decreased in day-2 (D2) as compared with day-0 (D0) of preadipocytes. Interestingly, we found an increasing trend in the expression level of *SH2B2* from D2 to day-10 of adipocytes differentiation.

### Biological evolution and conservation of SH2B2

**Figure 3:**
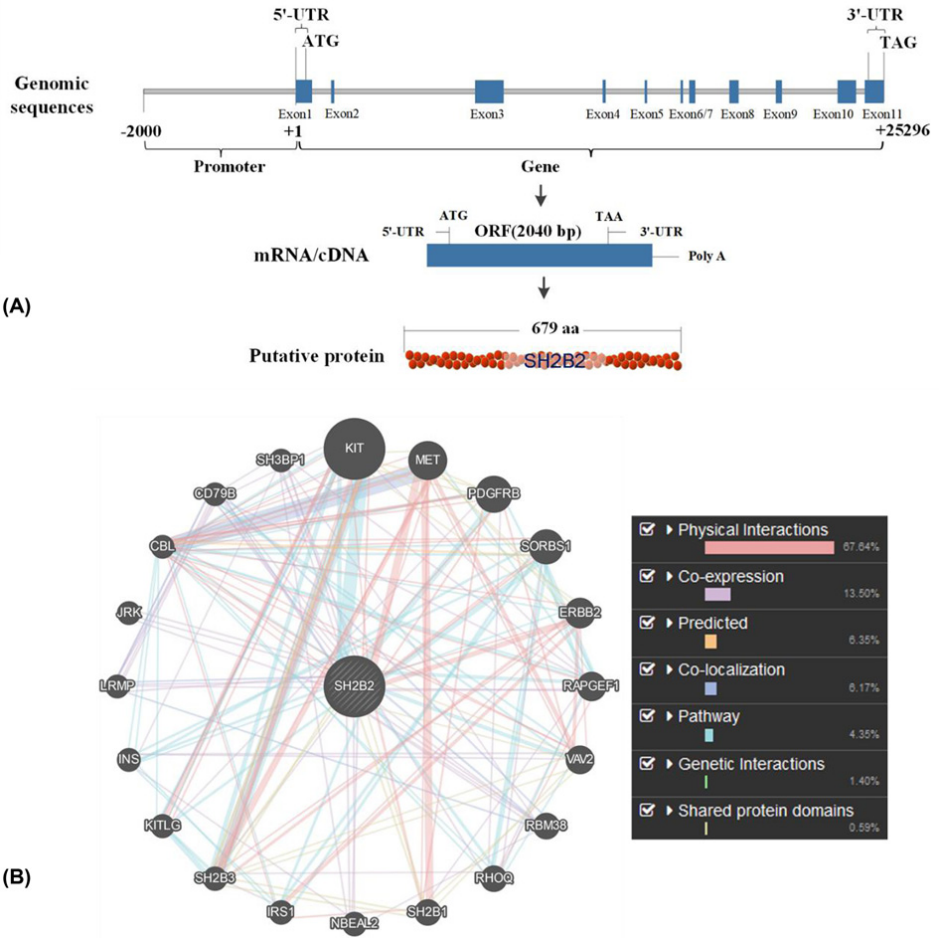
Structure and genetic interaction of *SH2B2* gene (A) molecular structure of SH2B2 gene, the source of information was (www.ncbi.nlm.nih.gov) (B) Genetic interaction of SH2B2 gene with other target genes

The SH2B2 gene is located on chromosome 25 of the bovine genome. The total length of *SH2B2* is 25296 bp, comprising the genomic coordinates starting from 34677735 to 34703030 (NC_037352.1, Reference genome bos taurus ARS-UCD1.2). This gene comprises 11 exons, the ORF which started from the start codon to the stop codon is 2040 bp, and the putative protein contains 679 amino acids (Figure 3A). The predicted network interaction among the *SH2B2* with other genes shows 67.64 % physical interactions. The co-expression, co-localization, and shared protein domains structures were 13.50%, 6.17 %, and 0.59%, respectively. (Figure 3B).

**Figure 4:**
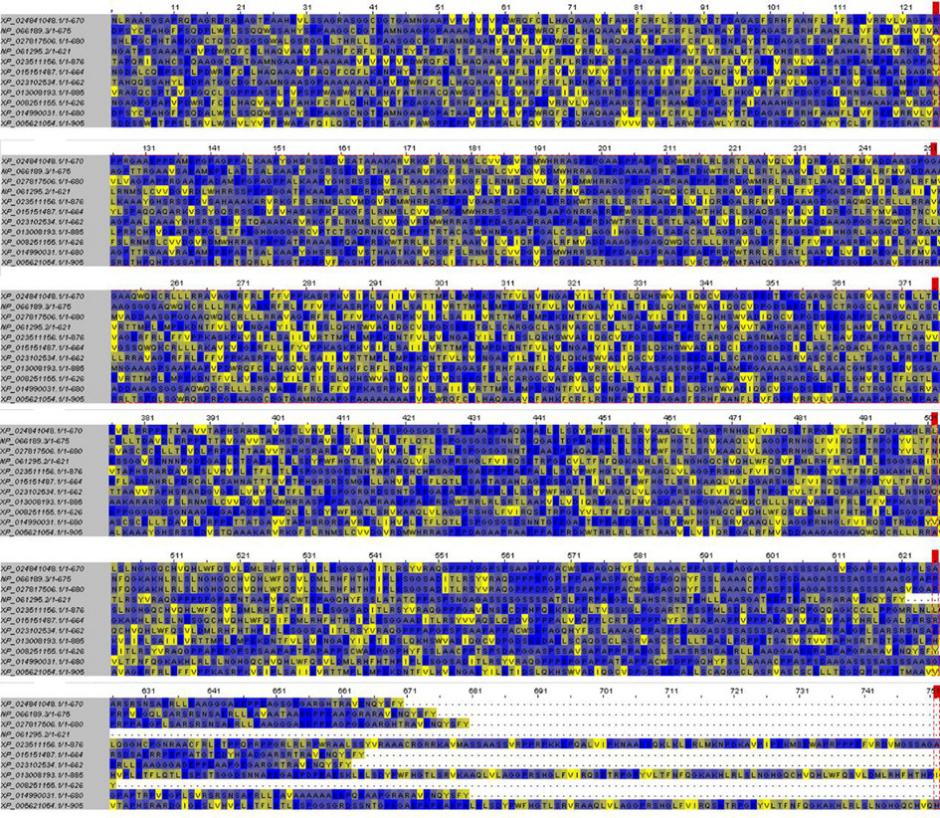
SH2B2 Protein Sequences (Multiple sequence alignment) of 11 species The conserved properties were marked with different background shading. With blue being 100%; gray with blue, 80%; gray with yellow, 60%, and white, not conserved.

**Figure 5:**
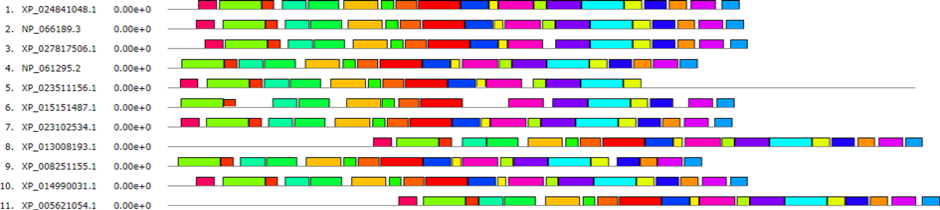
Conserved structural motifs of 11 species The *P*-value shows the significance of the motif site. The length of the color block shows the position, strength and significance of a particular motif site. The motif sites length is proportional to the negative logarithm of the p-value of the motif site. These colors are given through motif analysis performed through MEME suit system.

**Figure 6:**
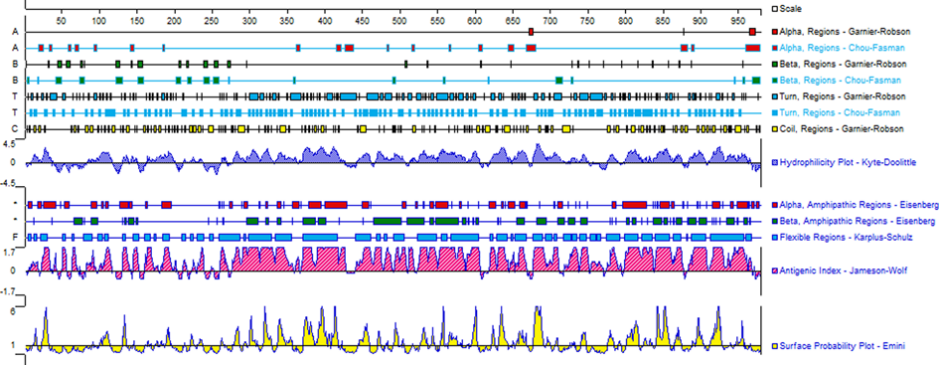
The prediction result of secondary structure in SH2B2 protein

**Figure 7:**
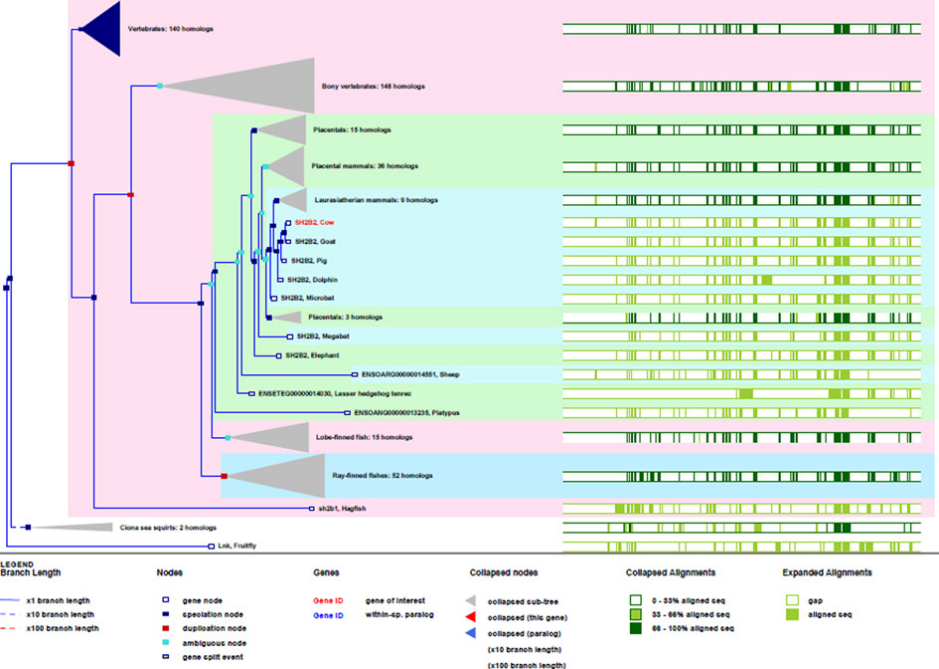
Detailed Phylogenetic tree of SH2B2 gene in different animals

**Figure 8:**
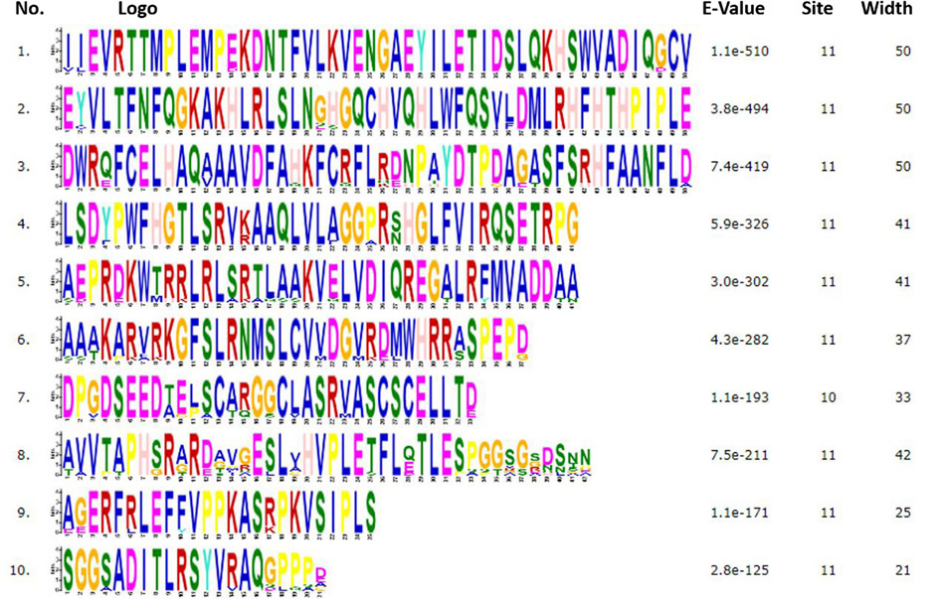
Ten significant SH2B2 protein motifs within 11 different selected species, identified through MEME suit The different colors within the motifs represent abbreviation of different amino acids.

The result of multiple sequence alignment there were 11 kinds of *SH2B2* protein aligned. The conserved properties were marked with different background shading. With blue being 100%; gray with blue, 80%; gray with yellow, 60%, and white, not conserved (Figure 4), the MEME online suit was used to find common significant motifs in the super secondary protein structure of the *SH2B2* gene in 11 target species (Figure 5). We found that there were many similar structures between bovine *SH2B2* and other species. The secondary structure of bovine *SH2B2* protein was predicted by using the Protean program in DNASTRAR 6.0 software. The online tool SWISS-MODEL was used to predict the tertiary structure of the protein, and the SH2B2 protein α-helix, β-sheet, and β-turn level were predicted. Regular curling and other structures. As shown in (Figure 6), the SH2B2 gene comparative genomics was searched through Ensmbl database (ensembl.org/Bos_taurus). Genomic alignment showed total 521 numbers of genes, with 454 numbers of speciation nodes, 35 numbers of duplication and 31 numbers of ambiguous genes. The *SH2B2* of cattle, goat had the closest phylogeny, and the SH2B2 of Elephant, Hagfish was much more distant from the bovine branch of the phylogenic tree (Figure 7). The domains hits *SH2B2* were found not conserved in mice species. While for the rest of the species, all domains hits were found conserved. Total 10 significant motifs were found among 11 species (Figure 8), which indicated that there is functional similarity among the selected species at the protein super secondary structure level.

## Discussion

Body measurement and carcass quality traits are used for the assessment of animals’ worth. The loin area muscle and intramuscular fat contents are the key indicators of meat quality grading. These traits are mostly affected by age of the animals, management conditions such as nutrition and by genetics of the animals. To get sustainable improvement in these traits of economic importance, selective breeding is one of the effective strategies, but it takes very long time to get efficient genetic gain due to longer generation interval in cattle. The candidate gene strategy is an efficient tool to measure association between genetic polymorphism and traits of economic importance in marker assisted selection [1].

Genetic polymorphisms are linked with traits of economic importance in livestock, because of their impact on the expression of relevant genes [35–37], e.g. single polymorphism in the STAT3 [37], SIRT3 [38], KLF3 [39] SIX1 [36], and SIX4 [40] genes impacted on body measurement and meat quality traits in Qinchuan cattle breed.

In the present study, four SNPs (snp1 g.20545A>G, snp2 g.20570G>A, snp3 g.20693T>C, and snp4 g.24070C>A) that were detected in the bovine SH2B2 gene coding sequence (CDS) region possibly affects body measurement traits (BMTs) and meat quality traits (MQTs). To reveal the linkage relationships among these four SNPs, the linkage disequilibrium (LD) between these four sites were estimated, which indicated that the *r*^2^ values ranged from 0.000 to 0.343. Based on the *D*′ and *r*^2^ values, three closely linked loci were revealed in the Qinchuan breed**.** According to an earlier research, if the value of *r* ^2^ is over 0.33, the LD is considered to be strong [41] Our result revealed that there was a strong linkage between g.20693T>C, and g.24070C>A, others linkages with pair-wise *r*^2^ < 0.33 were of weak kind.

In the present study, we found significant associations of genotypes g.20570G>A and g.24070C>A, with body measurement and carcass quality traits. Here, both g.20570G>A and g.24070C>A were located in the intron region and did not change the structure of the encoded proteins, but our results demonstrated that it was still associated with several growth traits. Such associations may be the result of linkage disequilibrium between this SNP and other genes on the same chromosome that have a significant effect on the growth traits studied here [42]. Another reason may be that mutations within introns could affect both the splice donor site or nearby regions and regulatory motifs within introns [43].

Thus, we further analyzed the effects of the combined genotypes above and growth traits in cattle. Haplotypes composed of SNPs could provide accurate information than single marker analysis for economic trait associations, due to the ancestral structure captured in the distribution of haplotypes. The Hap1 (–AATC–) had the highest haplotype frequencies (33.70%). The probable cause could be artificial selection in the Qinchuan cattle population, particularly the genomic regions influencing traits of economic importance [44,45]

Moreover, to further exploit the function of the *SH2B2* gene in the growth and development of Qinchuan cattle, mRNA expression was investigated in different tissues and adipocytes of Qinchuan cattle. Highest expression was found in small intestine, muscle, and fat. These findings show the role of SH2B2 in metabolism, growth and development, which are supported by the previously published literature, and that SH2B2 is a positive regulator of energy and glucose metabolism [46]. In addition, we also found high expression of SH2B2 in proliferation stage of preadipocytes, which was then slightly decreased in differentiation stage of day 2, and then an increasing trend was found in the expression level of SH2B2 from day 2 to 10 of adipocytes differentiation. These findings show role of SH2B2 in proliferation and differentiation of bovine adipocytes in Qinchaun cattle. Our results are in line with the findings of previously published literature [14]. Similarly, a previous study reported that g.1220C>T and g.21049C>T showed significant associations with body weight, average daily gain, body height, body length, and hucklebone width of Nanyang cattle at different ages [47].

## Conclusion

In conclusion, association analysis between SH2B2 gene polymorphisms indicated that g.20570G>A, g.20693T>C, and g.24070C>A, significantly associated with growth traits in Qinchuan cattle. In addition, H4H3 and H5H5 diplotype had highly significantly (*P* < 0.01) greater body length (BL), rump length (RL), and chest circumference (CC) than H4H2. Our investigation will not only extend the spectrum of genetic variation of bovine SH2B2 gene, but also provide useful information for the marker assisted selection in beef cattle breeding program.

## Abbreviations

HWE: Hardy–Weinberg equilibrium
JAK2: Janus kinase-2
MAS: marker-assisted selection
*SH2B2*: Src homology 2 B 2
SNP: single-nucleotide polymorphism
